# TA-GC cloning: A new simple and versatile technique for the directional cloning of PCR products for recombinant protein expression

**DOI:** 10.1371/journal.pone.0186568

**Published:** 2017-11-01

**Authors:** Athanasios Niarchos, Anastasia Siora, Evangelia Konstantinou, Vasiliki Kalampoki, George Lagoumintzis, Konstantinos Poulas

**Affiliations:** Department of Pharmacy, University of Patras, GR, Patras, Greece; Indian Institute of Science, INDIA

## Abstract

During the last few decades, the recombinant protein expression finds more and more applications. The cloning of protein-coding genes into expression vectors is required to be directional for proper expression, and versatile in order to facilitate gene insertion in multiple different vectors for expression tests. In this study, the TA-GC cloning method is proposed, as a new, simple and efficient method for the directional cloning of protein-coding genes in expression vectors. The presented method features several advantages over existing methods, which tend to be relatively more labour intensive, inflexible or expensive. The proposed method relies on the complementarity between single A- and G-overhangs of the protein-coding gene, obtained after a short incubation with T4 DNA polymerase, and T and C overhangs of the novel vector pET-BccI, created after digestion with the restriction endonuclease BccI. The novel protein-expression vector pET-BccI also facilitates the screening of transformed colonies for recombinant transformants. Evaluation experiments of the proposed TA-GC cloning method showed that 81% of the transformed colonies contained recombinant pET-BccI plasmids, and 98% of the recombinant colonies expressed the desired protein. This demonstrates that TA-GC cloning could be a valuable method for cloning protein-coding genes in expression vectors.

## Introduction

Expression of recombinant proteins using various expression systems has extensive applications in different branches of life sciences. Recombinant proteins usually display high expression levels and can also be expressed in fusion with amino (N) or carboxyl (C)-terminal tags, facilitating the subsequent purification and analysis steps [1, 2]. The cloning of genes into protein expression vectors has its own particular requirements. First, the insertion of genes has to be directional for proper expression. Moreover, the ability of easy insertion of one protein-coding gene into a variety of vectors is particularly useful in order to define the most appropriate construct for the expression of a specific protein [3, 4].

For the cloning of protein-coding genes into expression vectors, three main ligase-dependent methods have been developed: directional cloning, TA cloning and blunt-end ligation. The directional cloning approach uses double restriction endonuclease digestion to create cohesive ends for both the gene insert and the vector [5]. The TA cloning method takes advantage of the A-overhangs created from Taq DNA polymerase amplification, or treatment at the 3΄-ends of the amplified DNA, for ligation in vectors carrying T-overhangs at their 3΄-ends [6]. In blunt-end ligation, the blunt ends are created on the DNA insert by proofreading DNA polymerase amplification or treatment, and then ligation into blunt ended vectors [7, 8].

*In vitro* ligase-independent cloning can be achieved using several different suitable methods. In these methods insert and vector sequences are flanked by common/overlapping nucleotide sequences and recombined. For example, the Seamless Ligation Cloning Extract technique uses bacterial extracts for recombination [9], while Sequence and Ligation-Independent Cloning utilizes the strong 3΄-5΄ exonuclease activity of T4 DNA polymerase to create cohesive ends between the insert and vector [10]. For the same purpose, exonuclease III [11] can be used. The Polymerase Incomplete Primer Extension cloning method [12] relies on incomplete PCR amplification of both the gene insert and the vector in order to produce the final PCR products with compatible cohesive ends. Also, the Circular Polymerase Extension Cloning technique utilizes PCR to form DNA inserts and linearized vectors into complete circular nicked plasmids [13]. Also, patented kits such as the Gibson Assembly [14], Gateway [15] and In Fusion [16] are among other commercially available kits suitable for the ligase-independent cloning of protein-coding genes in protein-expression vectors.

The aforementioned approaches for cloning have been used worldwide and have contributed extensively to the study of proteins, however they still have their own disadvantages. For instance, directional cloning using restriction endonucleases and ligase is a relatively long process incorporating multiple steps, including digestion of DNA insert and vector with endonucleases, that should not cut inside the sequence of the insert. Moreover, the enzyme-digested protein-coding gene can only be inserted into vectors carrying specific compatible ends. In TA and blunt-end ligation methods the gene inserts are cloned into the vectors in a bidirectional fashion; so 50% of all positive colonies are in the wrong orientation. Efforts then have to be applied during the colony screening process, in order to determine the colonies carrying the correctly orientated protein-coding gene. Ligase-independent cloning methods are inherently inflexible because they demand separate PCR amplifications of both the protein-coding genes and the linear vectors with different pairs of long primers (>30 bases) for cloning into each expression vector. Additionally, commercial patented ligase-independent cloning kits come at elevated costs.

## Aim of the study

**Fig 1:**
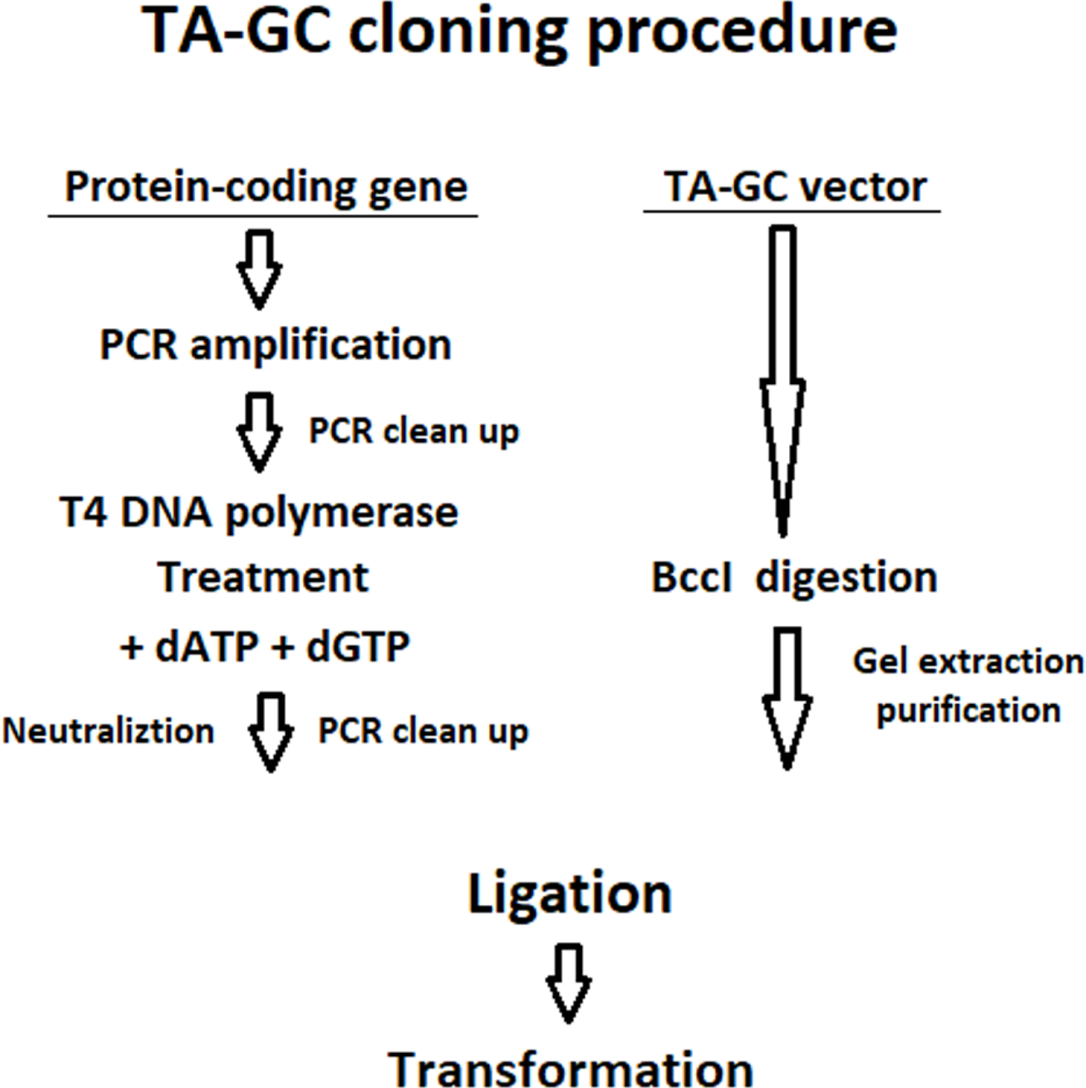
The procedure of TA-GC cloning. The target protein-coding gene is initially amplified using PCR and the PCR product is treated with T4 DNA polymerase. In parallel, the vector is digested with BccI. Afterwards, a ligation reaction between the purified linearized vector and the protein-coding gene is set up followed by transformation in high efficiency chemocompetent *E*. *coli* cells.

**Fig 2:**
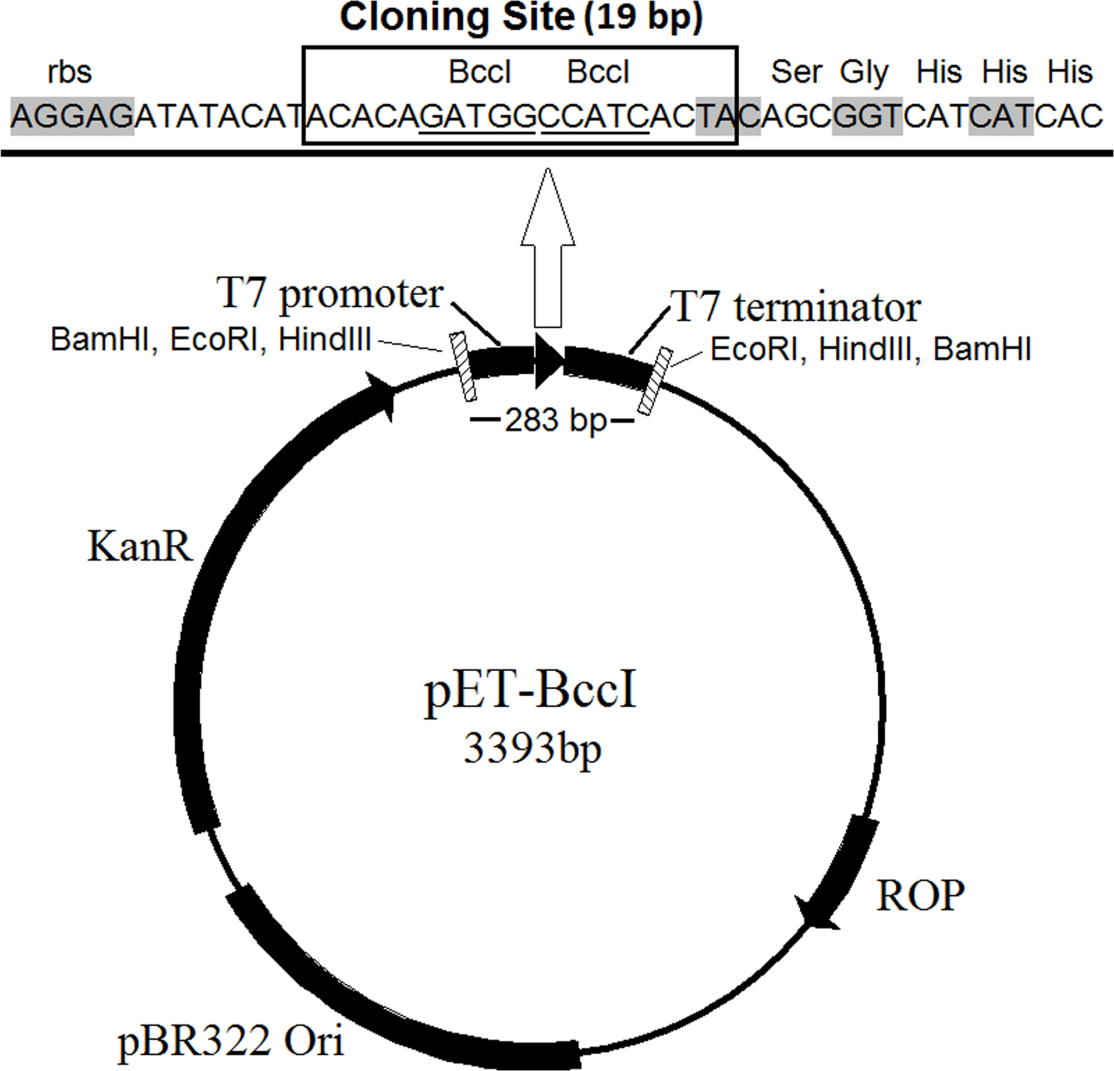
The novel protein-expression vector pET-BccI. The pET-26b (+) derived plasmid has a pBR322 origin of replication, which together with the ROP protein regulates the plasmid copy number per bacterial cell. The kanamycin resistance gene enables positive selection of the transformed *E*. *coli* cells in the presence of kanamycin. BamHI, EcoRI and HindIII recognition sites, flanking both sites of the T7 promoter, cloning site and T7 terminator cassette, facilitate the screening of the transformed colonies for the recombinant transformants. The cloning site of pET-BccI, composed of two adjacent reverse BccI recognition sites, provides single 5΄-T and C overhangs after digestion with BccI, which are suitable for the ligation of DNA molecules with complementary edges.

**Fig 3:**
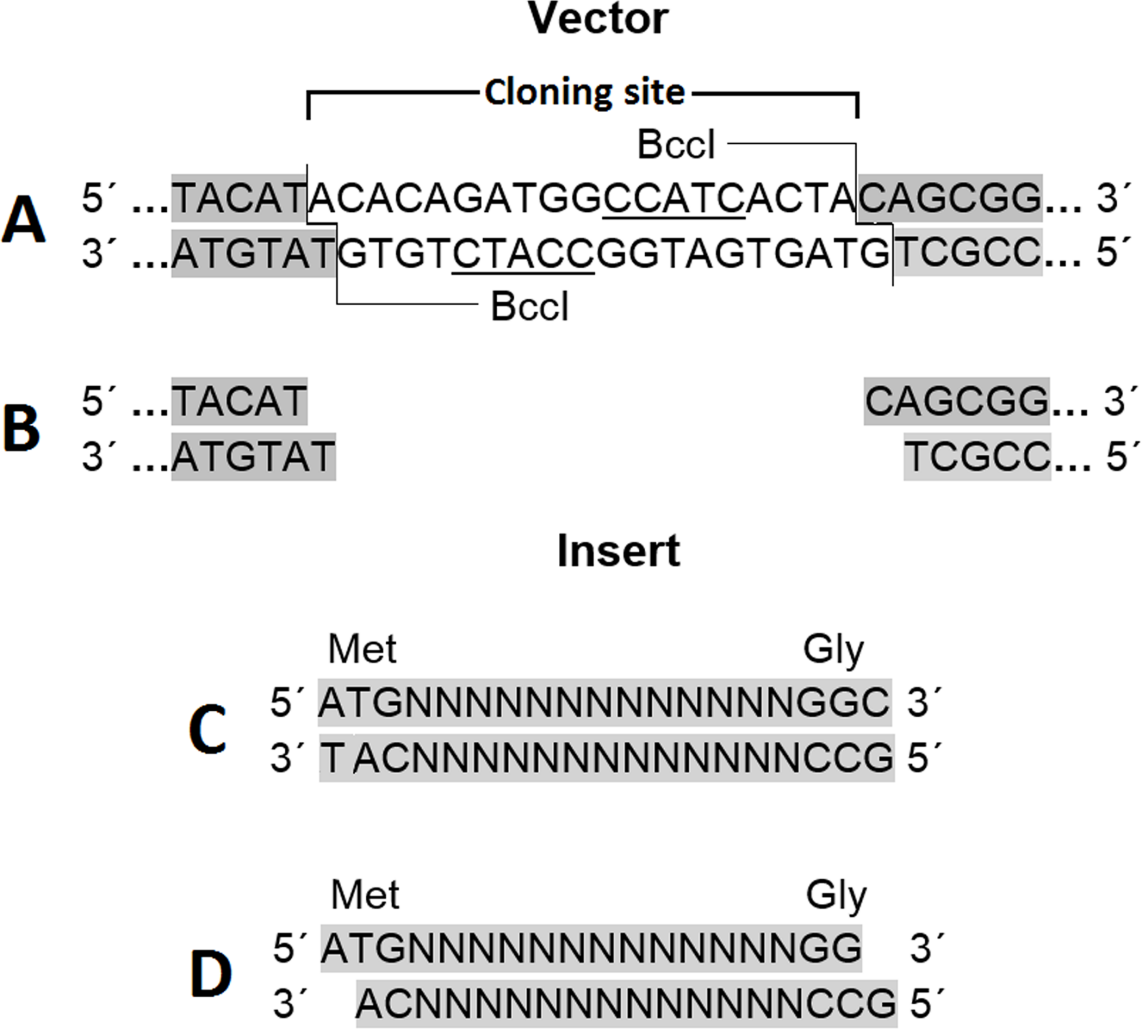
TA-GC cloning. **A**: BccI recognizes the sequence CCATC at the cloning site of pET-BccI and cuts out at the recognition site as indicated. **B**: after digestion with BccI. **C**: the protein-coding gene (starting with an ATG codon and always having a 3΄ glycine-coding GGC codon) after PCR amplification, and **D**: after treatment with T4 DNA polymerase in the presence of dATP and dGTP. The 5΄-Α and G overhangs of the protein-coding gene created after incubation with the T4 DNA polymerase are complementary to the 5΄-T and C overhangs of the pET-BccI vector after digestion with BccI.

In order to overcome the disadvantages of the existing cloning methods, herein, a new and simple cloning technique is proposed, named TA-GC cloning (**Fig 1**), which enables directional insertion of any protein-coding gene, in any compatible linearized vector, like pET-BccI (**Fig 2**), as demonstrated in this study. Protein-coding genes, flanked with an ATG methionine codon (**Fig 3**), or other appropriate adenine-started codons in cases of N-terminal tag expressing vectors, and a GGC glycine codon (**Fig 3**), or other appropriate cytocine-ended codons, can be cloned using the proposed method. If desired, a stop codon before the 3’- end of the target gene can be added, for example in case the vector does not have a stop codon, or in case one does not want to express the C-terminal tag of a vector. After PCR amplification and a short T4 DNA polymerase treatment, protein-coding genes obtain single 5- A and G overhangs, complementary to the 5΄- T and C overhangs of a linearized vector, like pET-BccI, after digestion with the restriction endonuclease BccI (**Fig 3**). BccI is a category B restriction endonuclease which recognizes the sequence CCATC and cuts out of the recognition site, 4 bases towards the 3’ direction at the main DNA strand and 5 bases towards the 5΄ direction at the complementary strand, thus creating single 5΄ nucleotide overhangs (New England BioLabs). Vectors like pET-BccI can be created from existing plasmids by replacing the original cloning sites with the cloning site of pET-BccI, while omitting the BccI recognition sites apart from those of the cloning site. The cloning site of pET-BccI is composed of two adjacent reverse BccI recognition sites (**Figs 2 and 3**). The pET-BccI vector, which is a derivative of pET-26b, also facilitates the screening of transformed colonies by using just one of the following restriction endonucleases: EcoRI, Hind III or BamHI. Alternatively, colony PCR can be used as a more straightforward and faster choice for colony screening. The presented TA-GC cloning method is fast, uses low cost reagents and provides the capability for every protein-coding gene to be cloned in every compatible linearized vector like pET-BccI, regardless of its sequence. *In vivo* amplification of the vector minimizes the possibility for mutation and hence, the vector is ready for subsequent ligation after only a single enzymatic digestion.

## Materials and methods

### pET-BccI construction

PCR amplification (Q5 master mix, New England BioLabs) was performed using the primers (Eurofins) CTGAGACTCGAGTGAGCGCAACGCAATTAATG (forward) and CCATACAATCGATAGATTGTCGCA (reverse) and 1 ng of pET-26b (Novagen) as the DNA template. Following this, 10 μg of PCR product was digested for 2 hours at 37°C with 10 units of the restriction endonucleases ClaI and XhoI (New England BioLabs) in NEB buffer 4. The same digestion reaction was set up using 10 μg of recombinant plasmid pUC 57 with synthetic DNA (Genscript). After electrophoresis and gel extraction purification (NucleoSpin^®^ Gel and PCR Clean-up, Macherey-Nagel) of both the digested synthetic DNA and the PCR product, 50 ng of the former and 100 ng of the latter were ligated using 400 units of T4 DNA ligase (New England BioLabs). The ligation reaction took place in 1 × T4 DNA ligase buffer, supplemented with 5% PEG 6000, at 25°C, for 1 hour. Then 5 μl of ligation reaction were used to transform 50 μl MAX Efficiency DH5α chemocompetent *E*. *coli* cells (Invitrogen), according to the manufacturer’s protocol, plated on Luria-Bertani (LB) plates with 30 μg/ml kanamycin, and then incubated 16 hours at 37°C. Four transformed colonies were picked up and grown in separate 5 ml liquid LB medium cultures supplemented with 30 μg/ml kanamycin after 14 hours incubation at 37°C under constant agitation at 250 rpm. Plasmid DNA was prepared using the NucleoSpin Plasmid purification kit (Macherey-Nagel), according to the manufacturer’s protocol. The purified pET-BccI batches were analyzed through the digestion of 1 μg plasmid DNA with 1 unit of the restriction endonucleases BccI, EcoRI, BamHI and HindIII (New England BioLabs), for 1 hour, at 37°C in the NEB buffers: CutSmart, EcoRI, 3.1 and 2.1, respectively.

### Cloning using TA-GC method

Initially the protein coding gene is amplified using primers that yield PCR product flanked by an ATG methionine codon and a GGC glycine codon (**Fig 3**), or as discussed in the aim of the study. The PCR product is purified and 1 μg of PCR product per 1kbp of product length is incubated in NEB buffer 2, with 1 unit of T4 DNA polymerase supplemented with 100 μM of dATP and dGTP, for 15 minutes at 12°C. The reaction is stopped by adding EDTA to a final concentration of 10 mM and heating at 75°C for 20 minutes. Afterwards, a PCR clean-up purification step is carried out. In parallel, 10 μg of the vector are digested with 10 units of BccI (New England BioLabs) in CutSmart buffer at 37°C for 1 hour, and subsequently subjected to electrophoresis and gel extraction purification. Thereafter, a ligation reaction between the purified linearized vector and the target gene is set up with a 1:2 vector/insert molar ratio, using 100 ng vector DNA and 400 units T4 DNA ligase. The ligation reaction is carried out in 1 × T4 DNA ligase buffer, supplemented with 5% PEG 6000, at 25°C, for 1 hour and 5 μl of ligation reaction are used to transform 50 μl of high efficiency chemocompetent *E*. *coli* (at least 1 x 10^7^ transformants/μg plasmid DNA) cells according to the standard protocols. The transformed *E*. *coli* cells are plated on Luria-Bertani (LB) plates with the appropriate antibiotic and then incubated 16 hours at 37°C. Then 4 colonies are randomly selected to be tested for recombinant plasmids.

In the current study in order to assess the novel vector pET-BccI, three protein coding genes were cloned and expressed. The bleomycin resistance protein (BRP), the chloramphenicol resistance (Chloramphenicol Acetyl Transferase, CAT) gene and the Classical Swine Fever Virus RNA Polymerase (CSFVRP) gene.

PCR amplification of the BRP gene (375 bp) was achieved using the primers ATGGCCAAGTTGACCAGTGCC (forward) and GCCGTCCTGCTCCTCGGCC (reverse) and 1 ng of plasmid pPICZaA (Invitrogen) as the template. Also, 1 ng of the plasmid pDEST14 (Invitrogen) was used as the template for the amplification of CAT gene (660 bp) using the primers ATGGAGAAAAAAATCACTGGATATACCAC (forward) and GCCCGCCCCGCCCTGCCA (reverse). Furthermore primers ATGGCATATGTTAAGCTAAGAGAGTTG (forward) and GCCCAGTGTCAGTTCTTCATAATGTT (reverse) were used for the amplification of CSFVRP gene (1830 bp), using 1 ng of the plasmid pUC 57 carrying synthetic CSFVRP gene (Genscript).

The ligation and transformation reactions followed the same procedure as described for the pET-BccI construction. The transformed *E*. *coli* cells were plated on Luria-Bertani (LB) 30 μg/ml kanamycin, and then incubated 16 hours at 37°C.

The above procedure (PCR amplifications, BccI digestion, ligations and transformations) was repeated three times and ten colonies from each transformation reaction were randomly selected for analysis. The selected colonies were used to inoculate 5 ml LB/kanamycin cultures, and after 14 hours incubation at 37°C under constant agitation at 250 rpm, plasmid DNA was purified as mentioned above. Then 1 μg DNA from every colony was digested with 1 unit of the restriction endonuclease BamHI in NEB buffer 2.1 at 37°C for 2 hours. Finally, the digestion reaction products were analyzed via electrophoresis. The presence of a 283 bp DNA band indicated there was no recombination, whereas the presence of a 283–19 bp plus the insert DNA band showed a successful recombination.

### Electrophoretic analysis of the DNA samples

The DNA samples were mixed with loading buffer containing 0.1% w/v Orange G and analyzed through horizontal electrophoresis in Tris-acetate-EDTA (TAE) buffer. One percent agarose (Nippon Genetics) gels were used for the separation of the DNA bands and the Nippon Genetics 100 base pair (bp) DNA Ladder was used as a molecular weight indicator.

### Expression test of recombinant pET-BccI

One Shot BL21(DE3) chemically competent *E*. *coli* bacteria (Invitrogen) were transformed, according to the manufacturer’s protocol, with 10 ng of each of the 26 pET-BccI plasmids carrying the BRP gene, 25 pET-BccI plasmids with the CAT gene and 22 pET-BccI plasmids carrying the CSFVRP gene. Two colonies from each transformation reaction were used to inoculate equal numbers of 3 ml LB/kanamycin pre-cultures, which were incubated overnight at 37°C and at 250 rpm. The following day, 15 ml cultures (LB/kanamycin) were inoculated with the appropriate volume from their corresponding pre-cultures in order to obtain an optical density (OD)_600_ value 0.1. The main cultures were incubated up to OD_600_ = 0.6 and then IPTG (Sigma) was added to a final concentration 1 mM. Four hours later, the cultures were centrifuged at 10,000 *g*, at 4°C for 5 minutes. The cell pellets were dissolved with 6 volumes of 1× SDS loading buffer and subjected to three instant freeze-thaw cycles from 95°C to -80°C and then finally stored at -18°C. Then 5 μl from each lysate were used for Western blot analysis after sodium dodecyl sulfate polyacrylamide gel electrophoresis. Also an equal volume of PiNK Prestained Protein Marker (Nippon Genetics) was used as a molecular weight indicator. The protein bands were electro-transferred to polyvinyl difluoride membranes (Macherey-Nagel) and, after blocking with 2% bovine serum albumin in phosphate buffered saline (PBS), a 1:2000 dilution of a mouse monoclonal anti-polyHistidine-Peroxidase antibody (Sigma) was used for detection of the expressed 6-Histidine (His) tagged proteins, followed by 3,3′-Diaminobenzidine (DAB) staining (500 μg/ml DAB (Sigma), 2 mM NiCl_2_ (Sigma) and 0.02% H_2_O_2_ (Sigma) in PBS).

### Statistical analysis

The percentages of the recombinant *E*. *coli* colonies obtained from the TA-GC cloning method were calculated as the ratio of the number of the colonies determined to be recombinant among the 90 randomly picked colonies from all three repetitions of the cloning experiments for the BRP, CAT and CSFVRP genes. The percentage of the protein expressing colonies in the presented method was calculated as the portion of the selected BL21 colonies transformed with the recombinant pET-BccI plasmids, which expressed BRP, CAT or CSFVRP proteins.

## Results

### pET-BccI vector construction

The novel vector pET-BccI (**Fig 2**) was constructed from the ligation of a major PCR-amplified fraction of pET-26b(+) (2269 bp) and a minor (1124 bp) synthetic DNA molecule. The resulting pET-BccI had a pBR322 origin of replication together with the Repressor of Primer (ROP) gene (**Fig 2**) (both elements from pET-26b(+)) for control of the plasmid copy number per bacterial cell at low levels. The kanamycin resistance gene enables positive selection of the pET-BccI transformed *E*. *coli*. The kanamycin resistance gene also originated from pET-26b(+), but it was modified by deleting three recognition sites for the BccI restriction endonuclease (2689 CCATC→AATGG, 2733 CCATC→CCATT, 3174 CCATC→CGATT). Also the BamHI, EcoRI and HindIII recognition sites, flanking upstream and downstream of the protein-expression cassette (i.e. the T7 promoter, the cloning site and the T7 terminator), facilitate screening for the recombinant pET-BccI (**Fig 2**). The cloning site of pET-BccI, composed of two adjacent reverse BccI recognition sites, provides single 5΄-T and C overhangs after digestion with BccI, which are suitable for the ligation of DNA molecules carrying single 5΄-Α and G overhangs (**Fig 3**). The proteins were expressed from pET-BccI with a C-terminal 6 × His tag through a Glycine-Serine-Glycine linker (**Fig 2**).

### Cloning using TA-GC method

Initially the protein coding gene is amplified using primers that give PCR product flanked by an ATG methionine codon and a GGC glycine codon (**Fig 3**), or as discussed in the aim of the study. The PCR product after incubation with T4 DNA polymerase supplemented with dATP and dGTP, obtains single 5’- A and G overhangs. Afterwards, a PCR clean-up purification step is carried out, while in parallel, vector is digested with BccI. After electrophoresis and gel extraction purification of the digested vector, a ligation reaction between the purified linearized vector and the protein-coding gene is set up followed by transformation in high efficiency chemocompetent *E*. *coli* cells.

### Assessment of pET-BccI

**Fig 4:**
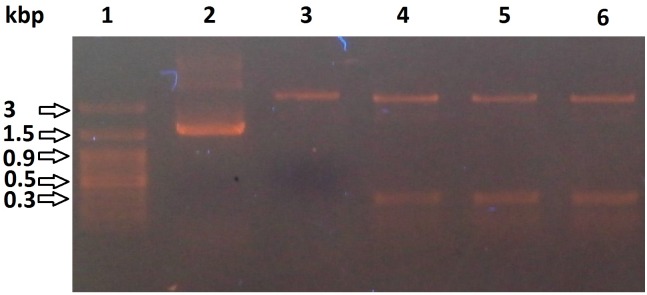
pET-BccI untreated and digested. **1**: DNA ladder. **2**: pET-BccI untreated. **3**: pET-BccI digested with BccI. **4**, **5**, **6**: pET-BccI digested with EcoRI, BamHI and HindIII, respectively.

The constructed pET-BccI was analyzed through electrophoresis following digestion using the restriction endonucleases BccI, BamHI, EcoRI and HindIII (**Fig 4**) and also through Sanger DNA sequencing. In order to assess the functionality of pET-BccI, BRP, CAT and CSFVRP genes, these were cloned in the plasmid and after transformation in competent *E*. *coli* bacteria the grown colonies were screened for cells containing a recombinant vector. Subsequently, pET-BccI constructs carrying the BRP, CAT or CSFVRP genes were tested for the expression of the corresponding proteins.

### Screening for recombinant pET-BccI

**Fig 5:**
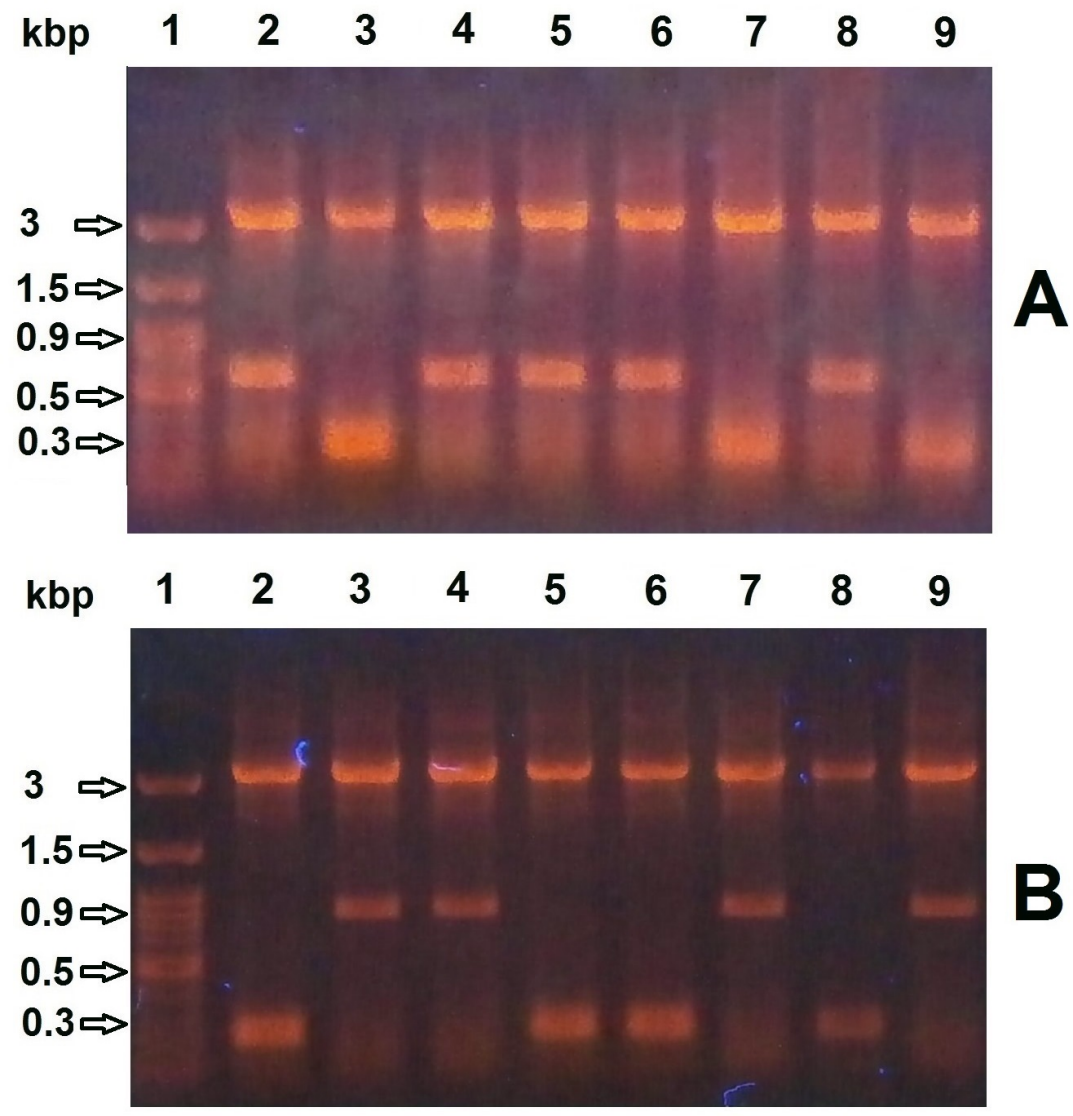
Screening for *E*. *coli* colonies transformed with recombinant pET-BccI. Plasmid preparations from cultures inoculated with transformed colonies were digested with BamHI and subjected to electrophoresis. **A**) For BRP recombinants pET-BccI screening, the presence of a 283 bp DNA band indicated there was no recombination (A3, A7, A9), whereas the presence of a 636 bp insert DNA band showed a successful recombination (A2, A4, A5, A6, A8). **B**) For CAT recombinants pET-BccI screening, again the presence of a 283 bp DNA band indicated there was no recombination (B2, B5, B6, B8), whereas the presence of a 924 bp insert DNA band showed a successful recombination (B3, B4, B7, B9).

The plasmid preparations from cultures inoculated with the transformed colonies were digested with BamHI and then subjected to electrophoresis. A 283 bp DNA band indicated that there was no recombination, whereas a 636 bp band (283–19 + 372 bp) for the BRP gene, or a 924 bp band (283–19 + 660 bp) for the CAT gene, or a 2094 bp band (283–19 + 1830 bp) for the CSFVRP gene, implied successfully recombined pET-BccI vectors (**Fig 5**). On average, the percentage of recombinant colonies was determined to be 81% (26/30 for BRP, 25/30 for CAT and 22/30 for CSFVRP) (Table 1).

**Table 1:** Raw number results for cloning efficiency of BRP, CAT and CSFVRP, and recombinant expression for the individual BRP and CAT genes.

Protein expressing gene	BRP	CAT	CSFVRP	Total
Gene size (bp)	375	660	1.830	** **
Transformed colonies tested	30	30	30	90
Recombinant colonies found	26	25	22	73
Colonies tested for protein expression	52	50	44	146
Protein expressing colonies	52	49	42	143
% RECOMBINANT COLONIES	81,111			
% PROTEIN EXPRESSING COLONIES	97,945			

### Protein expression with recombinant pET-BccI

**Fig 6:**
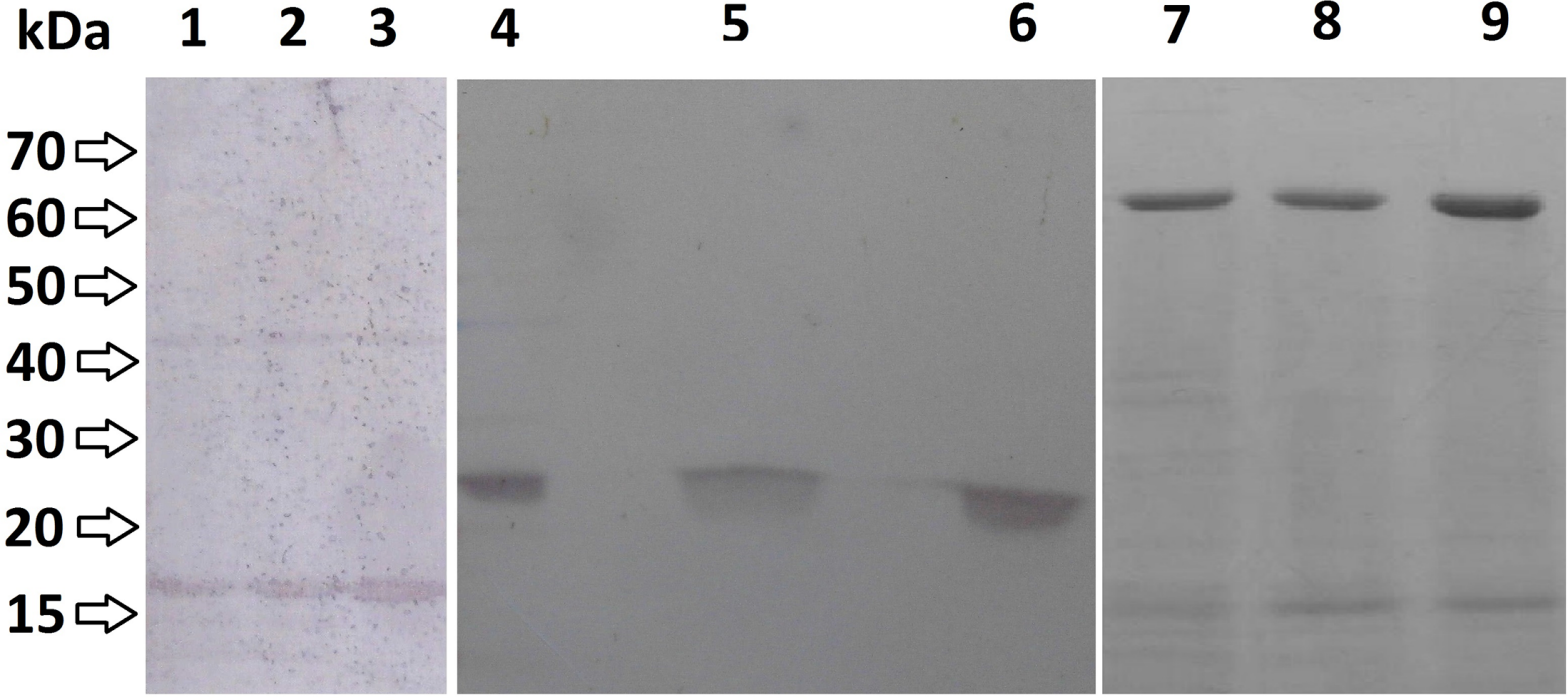
Western blot analysis of the expressed BRP, CAT and CSFVRP proteins. BL21 *E*. *coli* transformed with recombinant pET-BccI/BRP or pET-BccI/CAT were lysed after protein expression and analyzed by Western blot. **1**, **2**, **3:** Lysates from cultures inoculated with colonies transformed with pET-BccI/BRP **4, 5, 6:** lysates from cultures inoculated with colonies transformed with pET-BccI/CAT and **7,8,9:** lysates from cultures inoculated with colonies transformed with pET-BccI/ CSFVRP.

Plasmid preparations from cultures inoculated with DH5α™ *E*. *coli* colonies transformed with BRP- CAT- and CSFVRP-recombinant pET-BccI were used for the transformation of BL21 competent *E*.*coli* cells, which subsequently expressed the three genes. After expression, the *E*.*coli* cells were lysed and the lysates were analyzed using Western blot analysis for the BRP and CAT proteins and NiNTA purification for CSFVRP protein (**Fig 6**). From the recombinant colonies, 98% on average expressed one of the three proteins (52/52 for BRP, 49/50 for CAT and 52/54 for CSFVRP) (Table 1). Furthermore, randomly selected recombinant pET-BccI colonies that expressed BRP, CAT or CSFVRP proteins were subjected to DNA sequencing analysis, which confirmed the integrity of the constructs.

## Discussion

Recombinant proteins have many applications. The cloning of protein-coding genes into expression vectors has to be directional, and requires testing a number of vectors for both efficient cloning as well as protein expression. In the present study, a new, simple and flexible method for the cloning of protein-coding genes into expression vectors is presented, which is based on the complementarity between the 3΄ recessed ends of DNA inserts created by the T4 DNA polymerase and the corresponding 5΄ overhangs of the vector, such as in pET-BccI (**Fig 3**), created by the restriction endonuclease BccI. The presence of dATP and dGTP in the reaction prevents the formation of byproducts from over-digestion of protein-coding gene by T4 polymerase, which would prevent the subsequent ligation process.

T4 DNA polymerase has been used in previous studies for the ligation of DNA molecules. For instance, Wang and coworkers [17] used it for the DNA blunting of PCR products generated by Taq DNA polymerase, along with polynucleotide kinase in order to perform blunt end ligation with linearized blunt vectors. In other studies [10, 18], the T4 DNA polymerase was used to create long 3΄ recessed ends (at least 12 nucleotides long) to DNA inserts and linearized vectors, in order to form recombinant nicked plasmids suitable for the transformation of competent *E*. *coli*. Furthermore, BsaI, another type IIS restriction enzyme, has been used in combination with T4 DNA polymerase for cloning [19]. In the current study the restriction enzyme BccI was preferred over BsaI because it leaves a single nucleotide overhang vs the 4 nucleotide overhang of BsaI. Although this fact leads to decreased ligation efficiency, it adds fewer extra amino acids flanking the target gene and requires shorter primers for the amplification of the gene of interest. Furthermore the single nucleotide overhang provides greater flexibility than the 4 nucleotide overhang when the gene of interest is to be cloned in multiple expression vectors compatible with the proposed method. Any protein-coding gene, flanked with an appropriate adenine-started codon and an appropriate cytocine-ended codon can be cloned using the proposed method in any linearized vector with 5΄- T and C overhangs. In the study of Chaudhary et al [19], the gene of interest was expressed with 3 extra amino acids at the N-terminal and 3 at the C-terminal end. Moreover, the target genes were amplified using primers with 7-base long additional sequences at the 5’ ends. In our study the target genes were expressed with only 1 extra amino acid at the C-terminal end and the amplification of the gene of interest was done using only a 3-base long additional sequence at the 5’ end of the reverse primer.

In our proposed method, due to the T-A and G-C complementarity between the 5΄ mononucleotide overhangs of the insert and the vector, the cloning is directional. Furthermore, in the vector pET-BccI, presented in the current study (**Fig 2**), the presence of three recognition sequences of the restriction endonucleases EcoRI, BamHI and HindIII, each one both upstream and downstream of the expression cassette, i.e. the T7 promoter, the cloning site and the T7 terminator, facilitates the screening of the transformed colonies for the recombinants. The screening is facilitated by the need for only one of the three endonucleases, which are the most inexpensive restriction endonucleases available. Alternatively, the screening could be done using colony PCR. The screening tests of the cultures transformed with pET-BccI DH5a *E*. *coli* colonies showed that the percentage of the recombinant colonies was 81%; this value is possibly slightly lower than expected. It appears likely that partially incomplete digestion of the vector would lead to recircularization of empty vector. A larger removable linker between the tandem BccI sites could improve efficiency with this method. Nevertheless, in the proposed cloning method, 98% of the recombinant colonies expressed protein, in contrast with the blunt-end and TA cloning methods, which in 50% of the transformed colonies, the protein-coding gene was inserted in the wrong direction [6, 7]. Another advantage of the proposed method in comparison to blunt-end ligation is that no double insertion of the protein coding gene can be happened in the vector. The 5’- A and G overhangs on the insert molecules do not allow homo-polymerization. Furthermore, dephosphorylation of the vector and phosphorylation of the insert are not necessary [6]. One issue of the proposed method which has room for improvements regards the treatment of the protein-coding gene with T4 DNA polymerase and the subsequent neutralization of the enzyme. In the presented method, this procedure demands a considerable duration of time (35 minutes). In future studies, efforts will be made to minimize the incubation of the protein-coding gene with T4 DNA polymerase (e.g. by temperature rising). Furthermore, the possibility of purifying the reaction mixture, after the polymerase treatment, without previous neutralization will be explored.

The presented TA-GC DNA cloning method for the insertion of protein-coding genes in expression vectors exhibits significant advantages over the other currently used molecular cloning techniques. With the TA and blunt-end cloning methods, the gene is inserted non-directionally, in contrast to the proposed method. Directional cloning using double restriction endonuclease digestion [5], apart from also being a labor-intensive method, leaves unnecessary nucleotides at the edges of the protein-coding gene, often leading to the coding of several unnecessary amino acid residues at the protein edges or between the expressed protein and its tag. Using the method proposed herein, proteins are not expressed with additional amino acid residues at their termini, except for a glycine at the C-terminal and an extra amino acid at the N-terminal, in the case of N-terminal tagged proteins. The presence of undesired amino acids at the protein edges can also be avoided using cloning methods that utilize the 3΄→5΄ exonuclease activity of T4 DNA polymerase or exonuclease III to create complementary 3΄ recessed ends between the inserts and the linearized vectors [11, 18, 20]. Alternatively, non-wanted extra amino acids can be avoided by using methods such as exonuclease and ligation-independent cloning (ELIC) [21] and PIPE [12] that do not require 3' exonuclease activity. Nevertheless, using these cloning strategies, in order to avoid extra amino acids, apart from the requirement to utilize long oligonucleotide primers (30–50 nucleotides) for the PCR amplification of the target genes or expression vectors, the ability to clone the same PCR products into different protein-expression vectors is very limited. This is because different expression vectors must have identical DNA sequences, exactly at the point where they are usually different, i.e. at the nucleotide sequences next to the edges of the protein-coding gene, which code the N or C-terminal protein-tags. In contrast, in the TA-GC cloning method, every protein-coding gene can be cloned into any vector with the cloning site of pET-BccI, digested with BccI. Furthermore, in contrast to many costly commercial patented kits such as Gibson Assembly, Gateway and In Fusion, the TA-GC cloning method uses only a few, standard, inexpensive, unpatented reagents, rendering it an even more attractive choice. Taking into account all the above-mentioned points, the TA-GC method offers advantages for the insertion of the PCR-amplified genes in expression vectors and, therefore, it could be a prime technique for cloning protein-coding genes in the field of protein expression.

## Supporting information

S1 File pET-BccI sequence. pET-BccI plasmid sequence.
(ODT)
