# Supplementary material for: TA-GC cloning: A new simple and versatile technique for the directional cloning of PCR products for recombinant protein expression
Source: PLoS One. 2017 Nov 1;12(11):e0186568. doi: 10.1371/journal.pone.0186568 (PMC5665511; doi:10.1371/journal.pone.0186568)
Supplement: Supplementary file 1 [file pone.0186568.s001.odt]

pET-BccI sequense

GGTACCCCATGGGGATCCGAATTCAAGCTTAGATCTCGATCCCGCGAAATTAATACGACTCACTATAGGGAGACCACAACGGTTTCCCTCTAGAAATAATTTTGTTTAACTTTAAGAAGGAGATATACATACACAGATGGCCATCACTACAGCGGTCATCATCACCACCACCATTAAGATCCGGCTGCTAACAAAGCCCGAAAGGAAGCTGAGTTGGCTGCTGCCACCGCTGAGCAATAACTAGCATAACCCCTTGGGGCCTCTAAACGGGTCTTGAGGGGTTTTTTGCTGAAAGGAGGAACTATATCCGGATGAATTCAAGCTTGGATCCCTTAAG CTCGAGTGAGCGCAAC GCAATTAATG TAAGTTAGCT CACTCATTAG GCACCGGGAT CTCGACCGAT GCCCTTGAGA GCCTTCAACC CAGTCAGCTC CTTCCGGTGG GCGCGGGGCA TGACTATCGT CGCCGCACTT ATGACTGTCT TCTTTATCAT GCAACTCGTA GGACAGGTGC CGGCAGCGCT CTGGGTCATT TTCGGCGAGG ACCGCTTTCG CTGGAGCGCG ACGATGATCG GCCTGTCGCT TGCGGTATTC GGAATCTTGC ACGCCCTCGC TCAAGCCTTC GTCACTGGTC CCGCCACCAA ACGTTTCGGC GAGAAGCAGG CCATTATCGC CGGCATGGCG GCCCCACGGG TGCGCATGAT CGTGCTCCTG TCGTTGAGGA CCCGGCTAGG CTGGCGGGGT TGCCTTACTG GTTAGCAGAA TGAATCACCG ATACGCGAGC GAACGTGAAG CGACTGCTGC TGCAAAACGT CTGCGACCTG AGCAACAACA TGAATGGTCT TCGGTTTCCG TGTTTCGTAA AGTCTGGAAA CGCGGAAGTC AGCGCCCTGC ACCATTATGT TCCGGATCTG CATCGCAGGA TGCTGCTGGC TACCCTGTGG AACACCTACA TCTGTATTAA CGAAGCGCTG GCATTGACCC TGAGTGATTT TTCTCTGGTC CCGCCGCATC CATACCGCCA GTTGTTTACC CTCACAACGT TCCAGTAACC GGGCATGTTC ATCATCAGTA ACCCGTATCG TGAGCATCCT CTCTCGTTTC ATCGGTATCA TTACCCCCAT GAACAGAAAT CCCCCTTACA CGGAGGCATC AGTGACCAAA CAGGAAAAAA CCGCCCTTAA CATGGCCCGC TTTATCAGAA GCCAGACATT AACGCTTCTG GAGAAACTCA ACGAGCTGGA CGCGGATGAA CAGGCAGACA TCTGTGAATC GCTTCACGAC CACGCTGATG AGCTTTACCG CAGCTGCCTC GCGCGTTTCG GTGATGACGG TGAAAACCTC TGACACATGC AGCTCCCGGA GACGGTCACA GCTTGTCTGT AAGCGGATGC CGGGAGCAGA CAAGCCCGTC AGGGCGCGTC AGCGGGTGTT GGCGGGTGTC GGGGCGCAGC CATGACCCAG TCACGTAGCG ATAGCGGAGT GTATACTGGC TTAACTATGC GGCATCAGAG CAGATTGTAC TGAGAGTGCA CCATATATGC GGTGTGAAAT ACCGCACAGA TGCGTAAGGA GAAAATACCG CATCAGGCGC TCTTCCGCTT CCTCGCTCAC TGACTCGCTG CGCTCGGTCG TTCGGCTGCG GCGAGCGGTA TCAGCTCACT CAAAGGCGGT AATACGGTTA TCCACAGAAT CAGGGGATAA CGCAGGAAAG AACATGTGAG CAAAAGGCCA GCAAAAGGCC AGGAACCGTA AAAAGGCCGC GTTGCTGGCG TTTTTCCATA GGCTCCGCCC CCCTGACGAG CATCACAAAA ATCGACGCTC AAGTCAGAGG TGGCGAAACC CGACAGGACT ATAAAGATAC CAGGCGTTTC CCCCTGGAAG CTCCCTCGTG CGCTCTCCTG TTCCGACCCT GCCGCTTACC GGATACCTGT CCGCCTTTCT CCCTTCGGGA AGCGTGGCGC TTTCTCATAG CTCACGCTGT AGGTATCTCA GTTCGGTGTA GGTCGTTCGC TCCAAGCTGG GCTGTGTGCA CGAACCCCCC GTTCAGCCCG ACCGCTGCGC CTTATCCGGT AACTATCGTC TTGAGTCCAA CCCGGTAAGA CACGACTTAT CGCCACTGGC AGCAGCCACT GGTAACAGGA TTAGCAGAGC GAGGTATGTA GGCGGTGCTA CAGAGTTCTT GAAGTGGTGG CCTAACTACG GCTACACTAG AAGGACAGTA TTTGGTATCT GCGCTCTGCT GAAGCCAGTT ACCTTCGGAA AAAGAGTTGG TAGCTCTTGA TCCGGCAAAC AAACCACCGC TGGTAGCGGT GGTTTTTTTG TTTGCAAGCA GCAGATTACG CGCAGAAAAA AAGGATCTCA AGAAGATCCT TTGATCTTTT CTACGGGGTC TGACGCTCAG TGGAACGAAA ACTCACGTTA AGGGATTTTG GTCATGAACA ATAAAACTGT CTGCTTACAT AAACAGTAAT ACAAGGGGTG TTATGAGCCATA TTCAACGGGA AACGTCTTGC TCTAGGCCGC GATTAAATTC CAACATGGAT GCTGATTTAT ATGGGTATAA ATGGGCTCGC GATAATGTCG GGCAATCAGG TGCGACAATC TATCGATTGT ATGGGAAGCC CGATGCGCCA GAGTTGTTTC TGAAACATGG CAAAGGTAGC GTTGCCAATG ATGTTACAGA TGAAATGGTC AGACTAAACT GGCTGACGGA ATTTATGCCT CTTCCGACCATTAAGCATTT TATCCGTACT CCTGATGATG CATGGTTACT CACCACTGCG ATCCCCGGGA AAACAGCATT CCAGGTATTA GAAGAATATC CTGATTCAGG TGAAAATATT GTTGATGCGC TGGCAGTGTT CCTGCGCCGG TTGCATTCGA TTCCTGTTTG TAATTGTCCT TTTAACAGCG ATCGCGTATT TCGTCTCGCT CAGGCGCAAT CACGAATGAA TAACGGTTTG GTTGATGCGA GTGATTTTGA TGACGAGCGT AATGGCTGGC CTGTTGAACA AGTCTGGAAA GAAATGCATA AACTTTTGCC ATTCTCACCG GATTCAGTCG TCACTCATGG TGATTTCTCA CTTGATAACC TTATTTTTGA CGAGGGGAAA TTAATAGGTT GTATTGATGT TGGACGAGTC GGAATCGCAG ACCGATACCA GGATCTTGCG ATTCTATGGA ACTGCCTCGG TGAGTTTTCT CCTTCATTAC AGAAACGGCT TTTTCAAAAA TATGGTATTG ATAATCCTGA TATGAATAAA TTGCAGTTTC ATTTGATGCT CGATGAGTTT TTCTAA GAATTAATTC ATGAGCGGAT ACATATTTGA ATGTATTTAG AAAAATAAAC AAATAGGGGT TCCGCGCACA TTTCCCCGAA AAGTGCCACC TG
